# Essential Role of the Zinc Transporter ZIP9/SLC39A9 in Regulating the Activations of Akt and Erk in B-Cell Receptor Signaling Pathway in DT40 Cells

**DOI:** 10.1371/journal.pone.0058022

**Published:** 2013-03-07

**Authors:** Masanari Taniguchi, Ayako Fukunaka, Mitsue Hagihara, Keiko Watanabe, Shinichiro Kamino, Taiho Kambe, Shuichi Enomoto, Makoto Hiromura

**Affiliations:** 1 Graduate School of Medicine, Dentistry, and Pharmaceutical Sciences, Okayama University, Okayama, Japan; 2 Multiple Molecular Imaging Research Laboratory, RIKEN Center for Molecular Imaging Science, Kobe, Japan; 3 Graduate School of Biostudies, Kyoto University, Kyoto, Japan; Okayama University, Japan

## Abstract

The essential trace element zinc is important for all living organisms. Zinc functions not only as a nutritional factor, but also as a second messenger. However, the effects of intracellular zinc on the B cell-receptor (BCR) signaling pathway remain poorly understood. Here, we present data indicating that the increase in intracellular zinc level induced by ZIP9/SLC39A9 (a ZIP Zrt-/Irt-like protein) plays an important role in the activation of Akt and Erk in response to BCR activation. In DT40 cells, the enhancement of Akt and Erk phosphorylation following BCR activation requires intracellular zinc. To clarify this event, we used chicken ZnT5/6/7-gene-triple-knockout DT40 (TKO) cells and chicken Zip9-knockout DT40 (cZip9KO) cells. The levels of Akt and ERK phosphorylation significantly decreased in cZip9KO cells. In addition, the enzymatic activity of protein tyrosine phosphatase (PTPase) increased in cZip9KO cells. These biochemical events were restored by overexpressing the human Zip9 (hZip9) gene. Moreover, we found that the increase in intracellular zinc level depends on the expression of ZIP9. This observation is in agreement with the increased levels of Akt and Erk phosphorylation and the inhibition of total PTPase activity. We concluded that ZIP9 regulates cytosolic zinc level, resulting in the enhancement of Akt and Erk phosphorylation. Our observations provide new mechanistic insights into the BCR signaling pathway underlying the regulation of intracellular zinc level by ZIP9 in response to the BCR activation.

## Introduction

Zinc is an essential trace element for living organisms and is contained in many proteins, such as zinc-finger-containing transcriptional factors and zinc-dependent metalloenzymes [1]. Therefore, dysfunctions of zinc homeostasis are currently known to be involved in the development of various diseases, such as cancer, inflammation, and diabetes [2], [3]. Two zinc transporter families, namely, the Zinc transporter (ZnT)/solute carrier 30a (Slc30a) family and the Zrt/Irt-like protein (ZIP)/solute carrier 39a (Slc39a) family, have been identified and characterized. There are nine members of the ZnT family and 14 members of the ZIP family, which tightly control cellular zinc homeostasis [4]–[6].

Recently, intracellular zinc has been established as a second messenger molecule in breast cancer cells [7], lymphocytes [8]–[10], and mast cells [11]. In cancer cells, ZIP7 induces the release of zinc into the cytosol and the resulting increased intracellular zinc level regulates the epidermal growth factor (EGF)/insulin-like growth factor (IGF) signaling pathway [12]. Regarding this signaling activation, it has been reported that ZIP7 is directly phosphorylated by casein kinase (CK2) [13]. Phosphorylation of ZIP7 leads to the release of zinc into the cytosol, leading to the activation of signaling factors, such as Akt and Erk. In addition, zinc has also been shown to affect the immune functions of the ZIP and ZnT families, including the enhancements of T cell receptor signaling and protein kinase C (PKC) signaling, and the regulation of production of cytokines such as interleukin-2 (IL-2) and interferon-gamma (INFgamma) [14], [15]. The alteration of ZIP6 expression by lipopolysaccharides (LPS) in dendritic cells decreases intracellular zinc level and induces dendritic maturation [16]. Moreover, the protein expression of ZIP8 is significantly induced in infectious diseases and inflammation, and ZIP8-mediated zinc transport into innate immune cells is important for proper immune function [17], [18]. Although many study have been reported that the intracellular zinc regulates signaling pathway in T cell and lymphocytes, however, the relationship of zinc and B cell receptor (BCR) signaling has been poorly understood.

BCR signal transduction affects the expression of metabolic genes or cytoskeletal proteins and leads to various cellular events such as the survival, growth, and apoptosis of B cells [19]–[21]. To clarify the molecular relationships among key signaling enzymes such as PI3K, Ras, and PLCgamma in the BCR signaling, DT40 chicken B cell lines have been utilized as a model [22]–[24]. Furthermore, the relationships between cellular zinc homeostasis and zinc transporters have been characterized using DT 40 chicken B cells [25]. ZnT5, ZnT6, and ZnT7 (ZnT5/6/7), which are located in the Golgi, incorporate intracellular zinc from the cytosol into the Golgi. These transporters are required in the loading of zinc to zinc-requiring enzymes, namely, alkaline phosphatases, for enzyme activation and are indispensable in homeostatic maintenance of secretory pathway function [26]–[29]. Furthermore, ZIP9 has also been identified and characterized as a resident protein in the Golgi in DT40 and HeLa cell lines [30]. However, the function of ZIP9 is not understood well.

We hypothesized that zinc released to the cytosol as induced by ZIP9 plays a pivotal role in the BCR signaling pathway. Thus, we examined the mechanisms underlying the activation of BCR signaling by intracellular zinc using cZip9KO cells established from the DT40 chicken B lymphocyte cell line, which has been used as a model to examine the significance of calcium in BCR signaling [31], [32]. First, by treating DT40 cells with an intracellular zinc chelator, *N,N,N′,N′*-tetrakis(2-pyridylmethyl) ethylenediamine (TPEN), we found that the levels of Akt and Erk phosphorylation decreased under BCR activation by an anti-IgM antibody. In addition, we observed that intracellular zinc contributes to the increase in phosphorylation levels in DT40 cells when intracellular zinc level was induced to increase using a zinc ionophore, zinc pyrithione (ZnPy). Second, we found that the phosphorylation of Akt and Erk triggered by the anti-IgM antibody or ZnPy treatment was significantly attenuated in cZip9KO cells. Under the same experimental conditions, the enzymatic activity of protein tyrosine phosphatase (PTPase) increased in cZip9KO cells. These biochemical events were restored by overexpressing hZip9 in cZip9KO cells. Finally, by fluorescence zinc staining, we detected that ZIP9 induced the release of zinc into the cytosol from the Golgi. The altered regulation of Akt and Erk in BCR signaling and PTPase activity in cZip9KO cells indicate that intracellular zinc regulates BCR signaling. Our observations provide new insights into the mechanism of Akt and Erk activation in the BCR signaling pathway, which underlies the regulation of intracellular zinc level by ZIP9.

## Materials and Methods

### Materials

1-Hydroxypyridine-2-thione zinc salt and 2,2′-dithiodipyridine were obtained from Sigma-Aldrich, Inc. (Saint Louis, MO, USA). *N,N,N′,N′*-Tetrakis(2-pyridylmethyl)ethylenediamine (TPEN) was obtained from Dojindo Molecular Technologies, Inc. (Kumamoto, Japan). Ionomycin, calcium salt was obtained from Invitrogen (Carlsbad, CA, USA). Newport Green PDX, FluoZin-3 and BODIPY TR-ceramide were obtained from Molecular Probes (Eugene, OR, USA). An anti-chicken IgM antibody (M4) was purchased from Beckman Coulter, Inc. (Fullerton, CA, USA). Specific antibodies against phospho-Ser473 Akt, Akt, phospho-Thr202/Tyr204 p44/42 mitogen-activated protein kinase (MAPK), and p44/42 MAPK, and hemagglutinin (HA)-Tag (C29F4) were purchased from Cell Signaling Technologies (Beverly, MA, USA). LY294002 as the PI3K inhibitor and U0126 as the MEK1/2 inhibitor were also purchased from Cell Signaling Technologies. Other chemical compounds were purchased from Nacalai Tesque (Kyoto, Japan).

### Cell culture, Treatment, Stimulation, and Protein Isolation

DT40 cell lines were cultivated in RPMI1640 supplemented with 10% fetal bovine serum (FBS), 1% chicken serum (ChS), and 50 µM 2-mercaptoethanol (2ME) at 39.5°C in a humidified atmosphere with 5% CO_2_. DNA was transfected into DT40 cells as described previously to disrupt c*ZnT5/6/7* and c*Zip9* [26], [27], and *hZip9*-HA was reintroduced as describe previously [30].

Exponentially growing DT40 cells were starved in RPMI1640 supplemented with 0.5% BSA and 50 µM 2ME at 37°C for 5 h. Cells were washed once with 0.5% BSA-HBSS (25 mM HEPES-NaOH, pH 7.4, 120 mM NaCl, 0.8 mM MgCl_2_, 5.4 mM KCl, and 2 mM CaCl_2_) and then resuspended in the same buffer at 3.5×10^5^ per 500 µL. The cells were stimulated with the anti-chicken IgM antibody or treated with ZnPy, ZnCl_2_, or Py at 37°C for 10 min or 30 min. In some experiments, the cells were preincubated with LY294002 as the PI3K inhibitor or U0126 as the MEK1/2 inhibitor at 37°C for 30 min before the treatment. The cells were collected and washed once with 0.5% BSA-HBSS and then lysed with a cell lysis buffer (50 mM Tris-HCl, pH 7.4, 150 mM NaCl, 1 mM EDTA, 1% Triton-X100, 0.1% SDS, 10 mM NaF, and 1 mM NaVO_3_) on ice for 15 min. Insoluble materials were removed by centrifugation, and protein level was measured by Bradford assay using BSA as the standard.

### Western blot analysis

Whole-cell lysate (30 µg protein) was resolved on 10% SDS-PAGE gels and separated proteins were transferred to nitrocellulose membranes. The membranes were blocked with 5% (w/v) nonfat skim milk in TBST (10 mM Tris-HCl, pH 7.5, 150 mM NaCl, 0.1% Tween 20) and immunoblotted with anti-phospho-Ser473 Akt (pAkt), anti-Akt (Akt), anti-phospho-Thr202/Tyr204 p44/42 MAPK (pErk), anti-MAPK (Erk), or anti-HA (HA) antibodies for overnight at 4°C in 5% BSA-TBST. After incubation, the membranes were washed three times with TBST and incubated for 1 h at room temperature with horseradish-peroxidase-conjugated secondary antibodies. The immunoreactive bands were visualized using Immobilon Western Chemiluminescent HRP substrates (Millipore, Billerica, MA, USA) and membranes were scanned using the Chemi-Doc XRS system (Bio-Rad, Hercules, CA, USA).

### Measurement of PTPase activity

Cells were resuspended in 2 ml of cold homogenizing buffer (0.25 M sucrose, 20 mM HEPES, and 1 mM EDTA) and homogenized with 20 strokes of a Dounce homogenizer. To remove the nucleus, the homogenate was centrifuged at 2,200 rpm for 10 min at 4°C. The post-nuclear supernatant was centrifuged at 12,000 rpm for 60 min at 4°C. The pellet containing cytoplasmic and organelle membranes, was lysed in PTP lysis buffer (50 mM Bis-Tris, 2 mM EDTA, pH 6.3 with HCl, 5 mM DTT, 20% glycerol, and 0.1% Triton X-100). Insoluble materials were removed by centrifugation (14,000 rpm for 5 min at 4°C), and protein level was measured by Bradford assay using BSA as the standard. 10 µg of lysate was preincubated at room temperature for 10 min. After the preincubation, 100 µL of substrate solution (10 mg/mL *p*-nitrophenyl phosphate in assay buffer: 50 mM Bis-Tris, 2 mM EDTA, pH 6.3 with HCl, and 5 mM DTT) was added to the lysate, which was further incubated at room temperature for 15 min. The level of *p*-nitrophenol produced was measured at an absorbance of 405 nm. PTPase activity was measured in terms of fold changes with respect to the activity of untreated WT cells as the control.

### Zinc-specific fluorescence staining and measurement of intracellular free zinc

Intracellular zinc staining was performed as described previously [29]. DT40 cells at 70% confluence were starved in RPMI1640 supplemented with 0.5% BSA and 50 µM 2ME at 37°C for 5 h. The cells were treated with 5 µM Newport Green PDX or FluoZin-3 for 30 min on coverslips coated with 0.05% poly-L lysine before treating them with ZnPy or the anti-IgM (M4) antibody for 10 min. To stain the Golgi, we used BODIPY TR ceramide (red fluorescence), and performed staining with Newport Green PDX or FluoZin-3. The cells were visualized by confocal fluorescence microscopy (Carl Zeiss LSM510, Germany). In addition, 5×10^5^ DT40 cells were subjected to the experiment of intracellular zinc measurement using Newport Green PDX. The concentration of intracellular free zinc was calculated from the mean fluorescence with the formula [Zn] = K_D_×[(F−F_min_)/(F_max_−F)]. F_min_ was determined by the addition of 10 µM TPEN, and F_max_ was determined by the addition of 75 µM ZnPy [33], [34]. For a calibration of these zinc measurements, we evaluated the amount of Newport Green PDX integrated into cells [33]. After treatment of Newport Green PDX (5 µM for 30 min), washed out with 0.5% BSA/HBSS, and then lysed with HBSS buffer containing 50 µM digitonin, and added excessive amounts of ZnSO_4_ (20 µM). The concentration of Newport Green PDX in DT40 cells was calculated using the calibration curve. Values are expressed as the mean ± standard deviations. Experiments and measurements were performed in triplicate independently.

### Statistical analyses

For multiple comparisons, one-way analysis of variance was performed followed by Bonferroni's Multiple Comparison Test using GraphPad Prism 5 software. Differences were considered significant at *P*<0.01.

## Results

### Activation of Akt and Erk phosphorylation by BCR activation requires intracellular zinc

**Figure 1:**
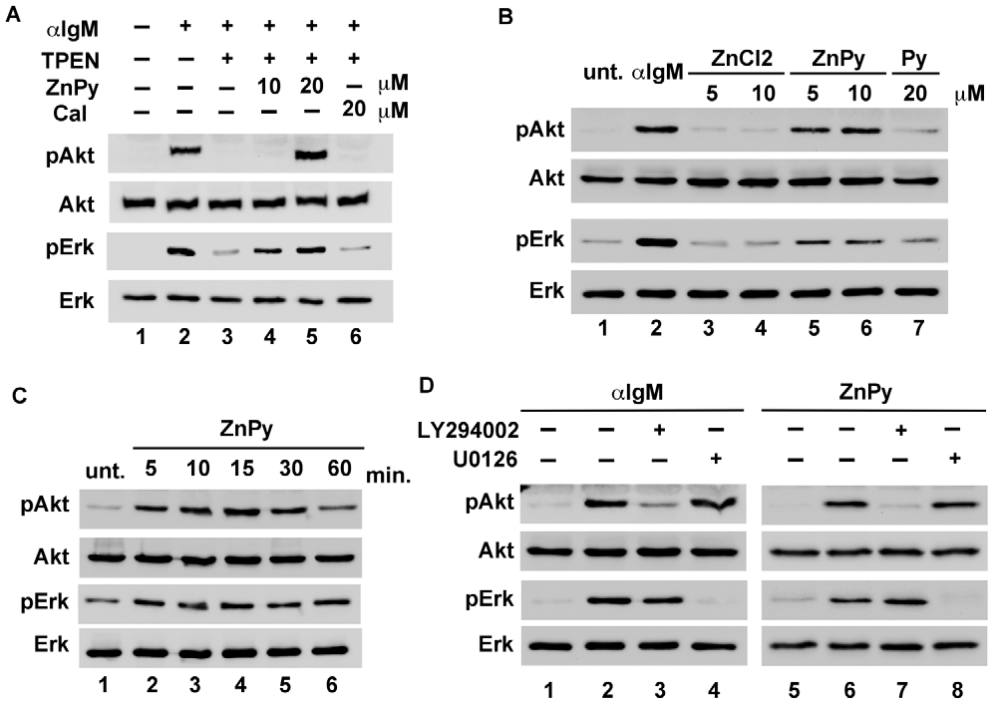
Effect of intracellular zinc on enhancement of Akt and Erk phosphorylation. (A) Enhancement of Akt and Erk phosphorylation required intracellular zinc. Serum-starved cells were treated with (+) or without (−) 10 µM TPEN for 1 h, before treatment with 0.5 mg/mL anti-IgM antibody (lane 2 and 3), and then treated with 10 and 20 µM ZnPy (lane 4 and 5) or 10 µM CaI (lane 6). (B) The treatment with ZnPy activated the phosphorylation of Akt and Erk. The abbreviation, “unt.” was defined the untreated sample. Serum-starved cells were treated with 0.5 mg/mL anti-IgM antibody (lane 2), 5 and 10 µM ZnCl_2_ (lanes 3 and 4), 5 and 10 µM ZnPy (lanes 5 and 6), and 20 µM pyrithione (lane 7) for 10 min. (C) Akt and Erk phosphorylation by ZnPy was enhanced in a time-dependent manner. The abbreviation, “unt.” was defined the untreated sample. Serum-starved cells were treated with 10 µM ZnPy for 5 min (lane 2), 10 min (lane 3), 15 min (lane 4), 30 min (lane 5), and 60 min (lane 6). (D) The inhibitors of PI3K and MEK1/2 inhibited the phosphorylation of Akt and Erk. Serum-starved cells were pretreated with (+) or without (−) LY294002 or U0126, before treatments with 0.5 mg/mL anti-IgM antibody (lanes 1–4) and 10 µM ZnPy (lanes 5–8). All data are representative of three independent experiments.

We first examined the impact of intracellular zinc level on the antigen-stimulated BCR signaling pathway. Western blot analysis was performed using lysates prepared from cells treated with or without the intracellular zinc chelator TPEN, using specific antibodies to total or phosphoforms of Akt and Erk (Figure 1A). We observed that the activation of BCR by the anti-IgM antibody induced the phosphorylation of Akt and Erk (Figure 1A, lane 2). Surprisingly, we found that the phosphorylation of Akt and Erk was not complete following the stimulation with the anti-IgM antibody when the cells were pretreated with 10 µM TPEN for 1 h (Figure 1A, lane 3). These suppressions were restored by the addition of 10–20 µM zinc pyrithione (ZnPy), but not recovered by calcium salt of ionomycin (CaI) (Figure 1A, lanes 4, 5 and 6).

To assess whether zinc is involved in the phosphorylation of Akt and Erk, DT40 cells were treated with zinc chloride (ZnCl_2_) and ZnPy at different concentrations and with the anti-IgM antibody. Figure 1B shows that the phosphorylation levels of Akt and Erk were significantly increased by 5–10 µM ZnPy as well as by the anti-IgM antibody (Figure 1B, lanes 2, 5, and 6). On the other hand, ZnCl_2_ and pyrithione (Py), which does not contain zinc, did not induce the phosphorylation of both proteins (Figure 1B, lanes 3, 4, and 7). We also determined whether ZnPy induces the phosphorylation of Akt and Erk in a dose-dependent manner. ZnPy enhanced the phosphorylation of both Akt and Erk in a dose-dependent manner, and Erk phosphorylation level was decreased by 25 µM ZnPy treatment (data not shown).

We next examined the time-dependence of the phosphorylation of Akt and Erk induced by ZnPy. The cells were treated with 10 µM ZnPy for the indicated durations to determine the optimal treatment time (Figure 1C). Akt phosphorylation by ZnPy was most enhanced by treatment for 10–15 min, but the enhancement was diminished at 60 min of treatment (Figure 1C, first panel; lanes 2–6). In contrast, Erk phosphorylation by ZnPy remained until 60 min. (Figure 1C, third panel; lanes 2–6).

To further investigate the phosphorylation of Akt and Erk by ZnPy treatment in DT40 cells, the cells were pretreated with LY294002 (PI3K inhibitor) or U0126 (MEK1/2 inhibitor) before treatment with the anti-IgM antibody and ZnPy. Figure 1D shows that the phosphorylation levels of Akt and Erk decreased in the presence of their respective inhibitors (Figure 1D, lanes 3 and 7 indicate LY294002, and lanes 4 and 8 indicate U0126).

Thus, the enhancement of Akt and Erk phosphorylation requires intracellular zinc and ZnPy, a zinc ionophore, which can solely activate the BCR signaling pathway.

### The phosphorylation of Akt and Erk is suppressed in the chicken Zip9-knockout DT40 cells

We examined whether the fluctuations of the levels of Akt and Erk phosphorylation are caused by the changes in intracellular zinc level, which was regulated by the zinc transporters. To study this mechanism, we used the two zinc-transporter-knockout cells, chicken *ZnT5/6/7* triple knockout (TKO) DT40 cells, and chicken *Zip9* gene knockout (cZip9KO) DT40 cells [26]–[30].

**Figure 2:**
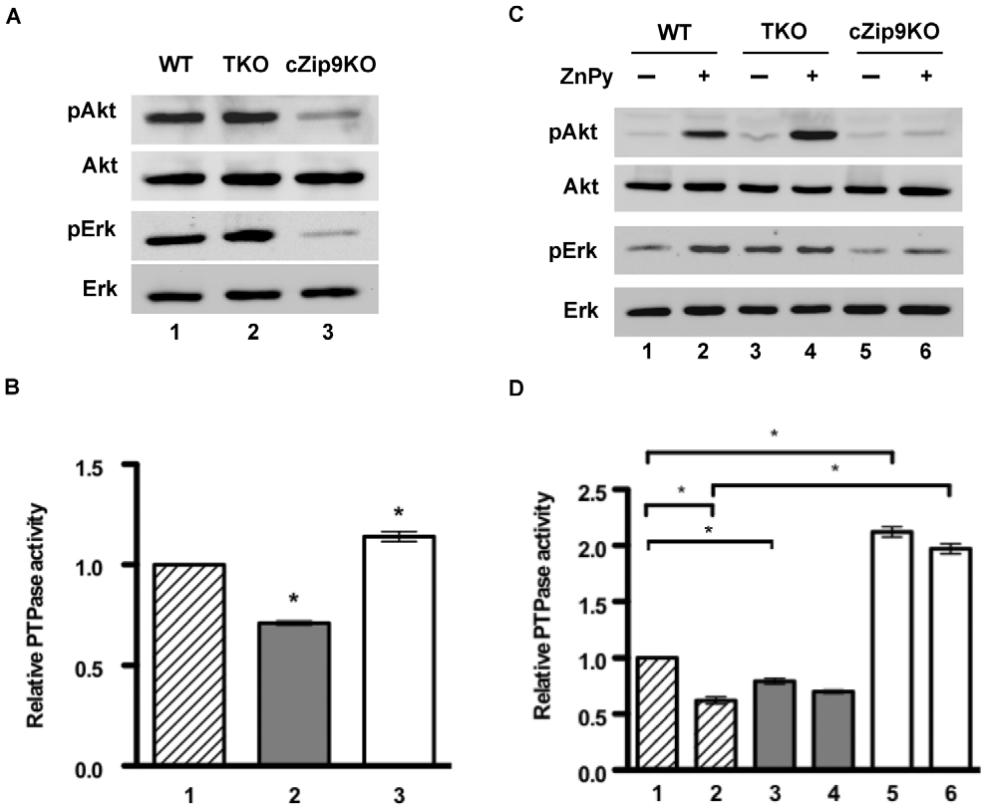
Akt and Erk phosphorylation in zinc-transporter-knockout DT40 cells. (A) Suppression of Akt and Erk phosphorylation in cZip9KO cells. Western blot analysis was performed using exponentially growing WT (lane 1), TKO (lane 2), and cZip9KO (lane 3) cells. (B) Analysis of total PTPase activity. WT (column 1), TKO (column 2), and cZip9KO (column 3) cells were subjected to PTPase assay. Values are expressed as the mean ± standard deviations. Significant difference at the level of *P<0.01 against the activity of WT cells (column 1). (C) ZnPy failed to induce Akt and Erk phosphorylation in cZip9KO cells. Serum-starved WT (lanes 1 and 2), TKO (lanes 3 and 4), and cZip9KO (lanes 5 and 6) cells were treated with (+) or without (−) 10 µM ZnPy for 10 min. (D) Analysis of PTPase activity in serum-starved DT40 cells. After treatment of serum-starved WT (columns 1 and 2), TKO (columns 3 and 4), and cZip9KO (columns 4 and 5) cells treated with (columns 2, 4 and 6) or without (columns 1, 3 and 5) 10 µM ZnPy for 10 min, and subjected to PTPase assay. Values are expressed as the mean ± standard deviations. Asterisk represents significant difference at the level of *P<0.01 for the columns linked by a line. All data are representative of three independent experiments.

We first examined the levels of Akt and Erk phosphorylation in these exponentially growing knockout DT40 cells in the presence of serum. The levels of Akt and Erk phosphorylation were identical in WT and TKO cells (Figure 2A, lanes 1 and 2). In contrast, surprisingly, the levels of Akt and Erk phosphorylation of cZip9KO cells significantly decreased (Figure 2A, lane 3). Under the same experimental conditions, we analyzed the activity of total protein tyrosine phosphatase (PTPase) using *p*-nitrophenol as a substrate. Figure 2B shows that the activity of PTPase decreased in TKO cells (gray column) as compared with that in wild-type (WT) cells (hatched column). On the other hand, the activity in cZip9KO cells moderately increased (white column).

To further investigate the phosphorylation levels of both proteins in TKO and cZip9KO cells, we examined whether ZnPy enhances the phosphorylation of Akt and Erk under serum-starved conditions. Figure 2C shows that the phosphorylation levels of Akt and Erk in WT and TKO cells were increased by the treatment with ZnPy (lanes 2 and 4). In TKO cells, the level of Erk phosphorylation did not decrease even under the serum-starved conditions (Figure 2C, third panel; lane 3). On the other hand, ZnPy did not increase the phosphorylation levels of Akt and Erk in cZip9KO cells (Figure 2C, lane 6). To confirm this observation, we also analyzed the activity of PTPase under the same experimental conditions. Figure 2D shows that the activity of total PTPase was decreased by the treatment with ZnPy in WT cells (hatched column 2). Total PTPase activity in ZnPy-treated TKO cells did not decrease significantly compared with that in non-ZnPy-treated TKO cells (gray columns 3 and 4). The activity of PTPase in non-ZnPy-treated TKO cells was slightly lower than that in WT cells (hatched column 1 and gray column 3). In contrast, the total PTPase activity in cZip9KO cells markedly increased (Figure 2C, white columns 5 and 6). These findings suggest that the regulation of intracellular zinc level by cZip9 in DT40 cells is required to increase the phosphorylation levels of Akt and Erk, at least in part, by inhibiting PTPase activity.

### Overexpression of human ZIP9 in cZip9KO cells restores the enhancement of Akt and Erk phosphorylation in response to zinc treatment and antigen-stimulated BCR

We demonstrated that cZip9 in DT40 cells function in the regulation of Akt and Erk phosphorylation. To clarify the effects of ZIP9 on both kinases, we overexpressed human Zip9 tagged with the hemagglutinin epitope (hZip9-HA) in cZip9KO cells and used the cells in the following experiments.

**Figure 3:**
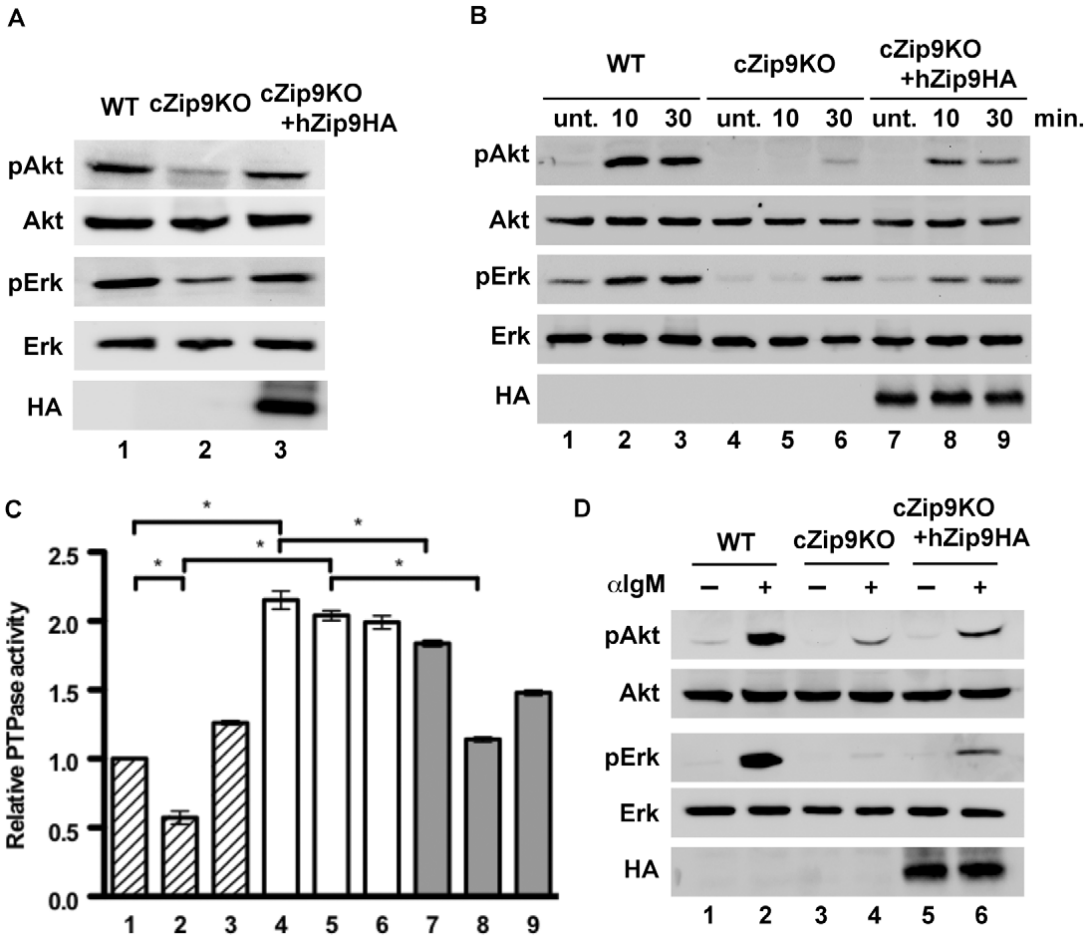
Effect of overexpression of human Zip9 on phosphorylation levels of Akt and Erk in response to zinc treatment and anti-IgM antibody stimulation. (A) Overexpression of hZip9 restored the phosphorylation of Akt and Erk. Western blot analysis was performed using exponentially growing WT (lane 1), cZip9KO (lane 2), and cZip9KO+hZip9HA (lane 3) cells. (B) Overexpression of hZip9 in cZip9KO cells by ZyPy treatment stimulated the phosphorylation of both proteins. Serum-starved WT (lanes 1–3), cZip9KO (lanes 4–6), and cZip9KO+hZip9HA (lanes 7–9) cells were treated with 10 µM ZnPy for 10 min (lanes 2, 5 and 8) and 30 min (lanes 3, 6 and 9). The abbreviation, “unt.” was defined the untreated sample. (C) Analysis of total PTPase activity. Serum-starved WT (lanes 1–3), cZip9KO (lanes 4–6), and cZip9KO+hZip9HA (lanes 7–9) cells were treated with 10 µM ZnPy for 10 min (lanes 2, 5 and 8) and 30 min (lanes 3, 6 and 9). Values are expressed as the mean ± standard deviations. Significant difference at the level of *P<0.01 for the columns linked by a line. (D) Overexpression of hZip9 restored the response to anti-IgM antibody-stimulated BCR activation. Serum-starved WT (lanes 1 and 2), cZip9KO (lanes 3 and 4), and hZip9-HA-overexpressing cZip9KO (lanes 5 and 6) cells were treated with 0.5 mg/mL anti-IgM antibody for 10 min. All data are representative of three independent experiments.

We re-estimated the phosphorylation levels of Akt and Erk by overexpressing hZip9-HA in exponentially growing cZip9KO cells in the presence of serum. As shown in Figure 3A, the overexpression of hZip9-HA in cZip9KO cells restored the phosphorylation of both proteins (Figure 3A, lane 3). In addition, the total PTPase activity in these cells was similar to that in WT cells under the same experimental conditions (data not shown). Under the serum-starved conditions, where we more closely examined the effects of ZnPy with time, we found that the phosphorylation of Akt was not enhanced in cZip9KO cells with ZnPy treatment for 10 and 30 min (Figure 3B, first panel; lanes 5 and 6) compared with that in WT cells (Figure 3B, first panel; lanes 2 and 3). Although the phosphorylation of Erk in cZip9KO cells was not enhanced after 10 min of ZnPy treatment, the phosphoform of Erk was detected following treatment with ZnPy for 30 min (Figure 3A, third panel; lanes 5 and 6). In contrast, hZip9-HA-overexpressing cZip9KO cells showed higher levels of the phosphoforms of both proteins than non-hZip9-HA-overexpressing cZip9KO cells (Figure 3B, lanes 8 and 9). ZnPy did not alter the protein level of hZip9-HA (Figure 3B, fifth panel; lanes 7–9). Under the same experimental conditions, we observed that the treatment with ZnPy inhibited the activity of total PTPase in the hZip9-HA-overexpressing cZip9KO cells compared with the non-hZip9-HA-overexpressing cZip9KO cells (Figure 3C, columns 5 and 8).

Figure 1A shows that the intracellular zinc chelation inhibits the phosphorylation of Akt and Erk in response BCR activation. Thus, we examined the effects of ZIP9 in response to anti-IgM antibody-stimulated BCR. Figure 3D shows that the phosphorylation levels of Akt and Erk did not increase in cZip9KO cells following the anti-IgM antibody treatment, in comparison with those in WT cells (Figure 3D, lanes 1–4). In contrast, the inhibitory effect on the phosphoforms of both kinases in cZip9KO cells was reversed by the overexpression of hZip9-HA (Figure 3D, first and third panels; lanes 4 and 6). The protein level of hZip9-HA was constant (Figure 3D, fifth panel; lanes 5 and 6).

Our experimental data indicate that ZIP9 plays an important role in the enhancement of Akt and Erk phosphorylation in response to the treatment with ZnPy and the anti-IgM antibody-activated BCR. In addition, the activation by the anti-IgM antibody significantly decreased the levels of phosphoforms of both proteins in cZip9KO cells. These observations coincide with the attenuation of Akt and Erk phosphorylation in TPEN-treated WT cells (Figure 1A).

### ZIP9 increases intracellular zinc level in response to BCR activation

**Figure 4:**
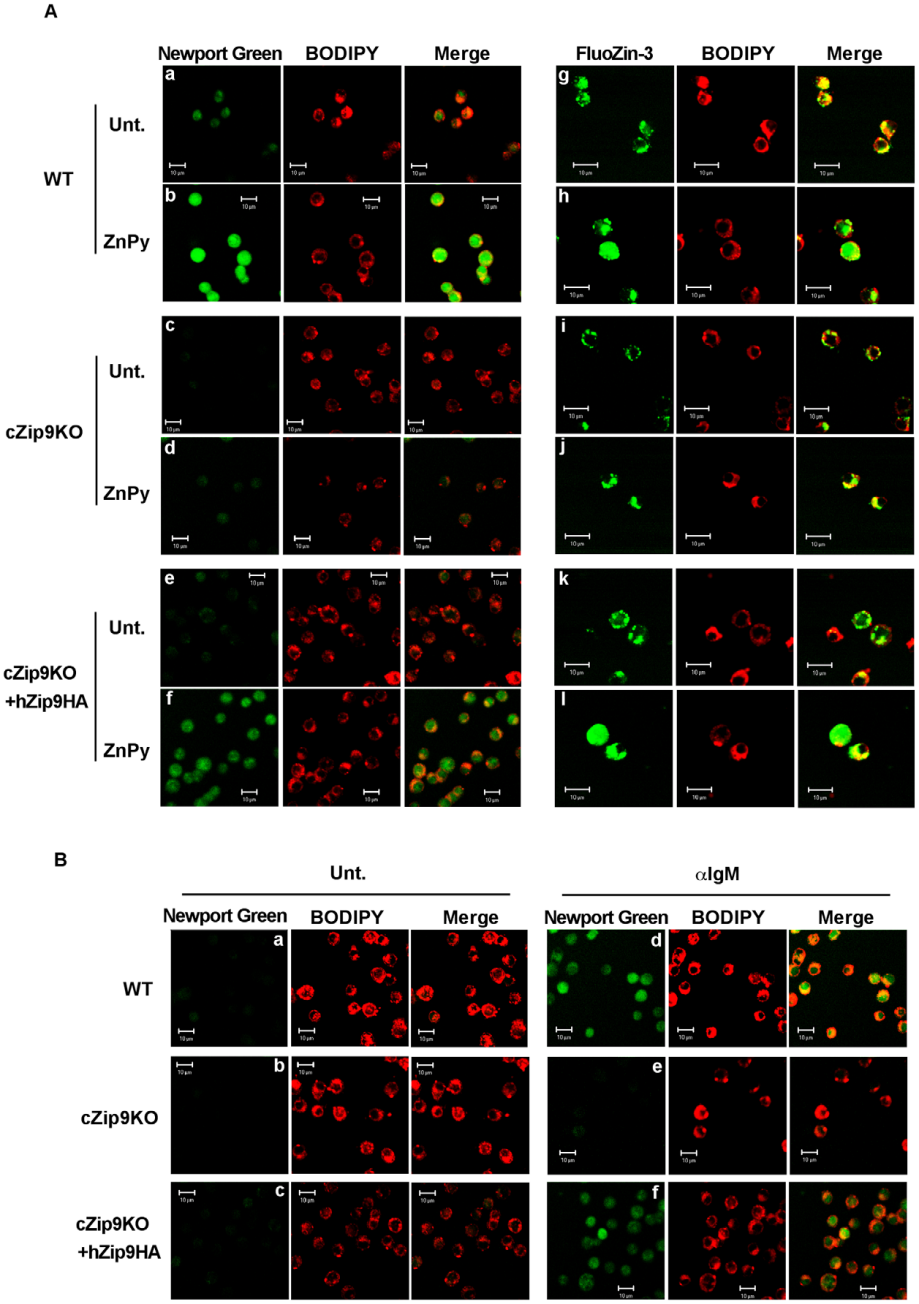
ZIP9 is an essential factor for regulating the intracellular zinc level in DT40 cells. (A) The intracellular zinc release depends on the expression of ZIP9. Serum-starved WT (panels a, b, g and h), cZip9KO (panels c, d, i and j), and hZip9-HA-overexpressing cZip9KO (panels e, f, k and l) DT40 cells were pretreated with 5 µM Newport Green PDX (magnification; ×40), FluoZin-3 (magnification; ×60) and BODIPY TR-ceramide for 30 min before treatment with 10 µM ZnPy (WT: panels b and h, cZip9KO: panels d and j, hZip9-HA-overexpressing cZip9KO: panels f and l) for 10 min. (B) Serum-starved WT (panels a and d), cZip9KO (panels b and e), and hZip9-HA-overexpressing cZip9KO (panels c and f) DT40 cells were pretreated with 5 µM Newport Green PDX (magnification; ×40) and BODIPY TR-ceramide for 30 min before treatment with 0.5 mg/mL anti-IgM antibody (WT: panel d, cZip9KO: panel e, hZip9-HA-overexpressing cZip9KO: panel f) for 10 min. The abbreviation, “unt.” was defined the untreated sample, and white bars were defined as 10 µm length.

A previous study demonstrated the presence of intracellular zinc around perinuclear regions and zincosome-like vesicles in cZip9KO cells using the zinc fluorescent probe zinquin [30]. It has been reported that zinquin recognizes mainly the zinc in subcellular compartments [35]. Hence, we performed the detection of intracellular zinc with confocal fluorescence microscope by using two types of zinc-specific fluorescent probes, Newport Green PDX (K_D_ = 30 µM) and FluoZin-3 (K_D_ = 15 nM) that have different distribution in the cells. As shown in Figure 4A, the fluorescence intensity of Newport Green PDX in WT cells treated with ZnPy was higher than that in untreated WT cells (Figure 4A, panels a and b). In contrast, we observed that the level of cytosolic zinc was not increased in cZip9KO cells by the ZnPy treatments (Figure 4A, panels c and d). On the other hand, the overexpression of hZIP9-HA restored the intracellular zinc contents in cZip9KO cells as well as WT cells, when the cells were treated with ZnPy (Figure 4A, panels e and f). In the staining with FluoZin-3, fluorescence intensity was increased by ZnPy treatment in WT and hZip9-HA-overexpressing cZip9KO cells as well as in Newport Green PDX staining. However, FluoZin-3 fluorescence of cZip9KO cells was localized at the Golgi, even if it was treated with ZnPy (Figure 4A, panels i and j).

When DT40 cells were treated with anti-IgM antibody, cytosolic zinc was also increased in WT cells (Figure 4B, panels a and d: Untreated WT cells = 2.05±0.17 µM and anti-IgM antibody stimulated WT cells = 2.87±0.30 µM). Whereas, intracellular zinc level in cZip9KO cells was lower than that in WT cells even if it was stimulated with anti-IgM antibody (Figure 4B, panels b and e: Untreated cZip9KO cells = 0.58±0.09 µM and anti-IgM antibody stimulated cZip9KO cells = 0.87±0.13 µM). In addition, hZip9-HA-overexpressing cZip9KO cells restore this decrease of cytosolic zinc, as well as treatment with ZnPy (Figure 4B, panels c and f: Untreated hZip9-HA-overexpressing cZip9KO cells = 1.88±0.18 µM and anti-IgM antibody stimulated hZip9-HA-overexpressing cZip9KO cells = 2.71±0.14 µM). We also observed that anti-IgM antibody stimulation for 5 min induced increase of intracellular zinc level in 5×10^5^ DT40 cells (Untreated cells = 2.05±0.17 µM, DT40 cells stimulated with anti-IgM antibody for 5 min = 2.61±0.22 µM, and stimulated for 10 min = 2.87±0.30 µM). These results suggested that intracellular zinc elevation by anti-IgM antibody appear prior to Akt and Erk phosphorylation, and there are sufficient amount of intracellular zinc for the activation of Akt and Erk after 5 min anti-IgM antibody stimuli. In addition, we demonstrated that more than 96.6% of Newport Green PDX had been incorporated in 5×10^5^ DT40 cells (WTcells = 4.89±0.80 µM, cZip9KO cells = 4.83±0.43 µM, and hZip9-HA-overexpressing cZip9KO cells = 4.90±0.46 µM).

Considering that ZIP9 is the Golgi-resident protein, these findings suggest that the increase in cytosolic zinc level is caused by zinc release from an intracellular store induced by ZIP9 through BCR activation. This observation is in agreement with the levels of Akt and Erk phosphorylation and the inhibition of total PTPase activity (Figure 3).

## Discussion

The essential trace element zinc is an important metal for all living organisms. Zinc is not only a nutrition factor, but it also functions as a second messenger [11]. Zinc homeostasis is tightly mainly regulated by two-types of zinc transporters, such as those belonging to the ZnT family and ZIP family. The latter zinc transporter family consists of 14 transporter proteins that are classified into four subfamilies (I, II, LIV-1, and gufA). Although the roles of ZIP family proteins have been extensively investigated and thus their importance has been elucidated [13], [17], [36], information available has been limited to that on the LIV-I and ZIP II subfamilies [25]. The cellular functions of ZIP9 belonging to the subfamily I have not been fully understood.

Intracellular zinc affects the immune functions of T-cells and lymphocytes including the activation of TCR signaling and cytokine production [8]. It has been reported that the increase in intracellular zinc level not only induces the activation of LCK and PKC [37], but also enhances the tyrosine phosphorylation of ZAP70, leading to the activation of the TCR signaling pathway [10]. The BCR signaling pathway is critical for many cellular events, such as cell growth, cell proliferation, and apoptosis [22]–[24]. BCR activation transduces the signal to several cascades, such as the PI3K-Akt, PLCgamma 2-PKC, and Ras-Raf-Erk cascades [24], [38], [39]. These cascades are important for the differentiation to antibody-producing cells and memory B cells. However, the effect of the intracellular zinc on the BCR signaling pathway remains unclear.

In this study, we examined the effect of intracellular zinc on the BCR signaling pathway activated by ZIP9 using the DT40 chicken B lymphocyte cell line as a model. Treating the cells with TPEN suppressed the Akt and Erk phosphorylation enhanced by BCR activation by the anti-IgM antibody. This suppression was reversed by supplementation of exogenous zinc into the cells (Figure 1A, lanes 4 and 5). In addition, the treatment of ZnPy without the anti-IgM antibody induced Akt and Erk phosphorylation in time- and concentration-dependent manners. Furthermore, the phosphorylation of both proteins was inhibited by the specific inhibitors of PI3K and MEK1/2. These observations indicate that intracellular zinc is necessary for the activation of the BCR signaling pathway. However, it has not been clarified whether zinc transporters are involved in the regulation of this signaling pathway.

ZIP9 has been suggested to function in the release of zinc from the Golgi to the cytosol. We investigated direct correlation between the effect of ZIP9 and the BCR signaling pathway. Interestingly, the phosphorylation of both Akt and Erk was suppressed by the disruption of *cZip9* in exponentially growing DT40 cells. This suppression was also observed in the serum-starved cells, even if they were treated with ZnPy or the anti-IgM antibody (Figures 2 and 3).

In TKO cells, the phosphorylation levels of Akt and Erk were similar to those in WT cells under the exponential growth condition (Figure 2A, lane 2). This finding raises the possibility that the accumulation of zinc in the Golgi is due to a ZnT5/6/7-independent mechanism. In contrast, the increase in the levels of these phosphoforms of proteins may be the result of inhibition of PTPase by cytosolic zinc, which is not incorporated into the Golgi. Further testing of these hypotheses is needed to clarify whether zinc transport into Golgi *via* ZnT5/6/7 is required for the function of ZIP9. In addition, the level of Erk phosphorylation did not decrease, even under the serum-starved condition. This finding seems to support the idea of sustained inhibition of PTPase by cytosolic zinc against the Erk cascade. The phosphorylation levels of Akt and Erk increased in the TKO cells as well as in WT cells under the ZnPy treatment conditions (Figure 2C). This finding suggests that ZnPy is incorporated in the Golgi [29] and then release from the Golgi *via* ZIP9, indicating that zinc trafficking mediated by cZIP9 is involved in the activation of the BCR signaling pathway.

Furthermore, we explored whether this activation is dependent on ZIP9, of which sequence is 89% identical to the cZIP9 protein [30]. Therefore, hZIP9-HA was overexpressed in cZip9KO cells to determine the effects of ZIP9. We observed that hZIP9-HA overexpression restored the enhancement of Akt and Erk phosphorylation in cells treated with ZnPy and the BCR signaling pathway treated with the anti-IgM antibody (Figures 3B and 3D).

We further explored whether the phosphorylation of Akt and Erk is regulated by PTPase. Our findings indicate that PTPase activity was significantly higher in cZip9KO cells than in WT cells (Figures 2D and 3C). In contrast, PTPase activity was decreased by overexpression of hZip9-HA in ZnPy-treated cZip9KO cells (Figure 3C). cZip9KO cells retained high activity of PTPase, even if cells were treated with ZnPy (Figure 3C, white columns 4, 5 and 6). These data indicate ZIP9 may inhibit PTPase activity.

We speculate two alternative explanations for the mechanism of PTPase inhibition by ZIP9. First, ZIP9 might elevate local zinc concentration on the Golgi where PTPase is in close proximity to ZIP9. Hence, liberated zinc from Golgi by ZIP9 could bind to the cysteine residue in the active site of PTPase and subsequently reduce its activity [40], [41]. Second, the released zinc could modulate protein stability. It has been reported that zinc inhibits PTEN activity in human airway epithelial cells [42] by zinc-induced its ubiquitin-dependent degradation [43]. As such, free zinc generated by ZIP9 could be involved in the proteolysis of PTPase through the ubiquitin-proteasome system.

Our findings suggest that the activation of the BCR signaling pathway following the treatment with ZnPy and the anti-IgM antibody is indeed the function of ZIP9. However, a question remains whether BCR activation increases the zinc influx through ZIP9 into the cytosol. It has been reported that the intracellular zinc level is altered by changes in the protein expression level of the zinc transporter [44] and the release of zinc from the ER in response to extracellular stimulation [11], [13]. We observed a significant fluorescence intensity of intracellular zinc in both exponentially growing WT and hZip9-HA-overexpressing cZip9KO cells (data not shown). In addition, we revealed the intracellular zinc distribution in DT40 cells that were serum-starved and treated with ZnPy or the anti-IgM antibody by using Newport Green PDX and FluoZin-3. These results showed that zinc is accumulated and retained into the Golgi in cZip9KO cells (Figure 4). We analyzed that Zip9 mRNA was ubiquitously expressed in the mouse tissues and several cell lines, such as HeLa, MCF7, and NIH3T3 cells (data not shown). One of them, we confirmed that treatment of ZnPy increased florescence intensity of zinc in the Golgi of Zip9 siRNA-treated HeLa cells (unpublished data). This supporting observation might indicate that ZIP9 release zinc from the Golgi to the cytosol in mammalian cells as well as DT40 cells.

It is known that zinc waves occur together with calcium waves [11], [13]. Antigen-stimulated BCR has been shown to trigger calcium release via the inositol 1,4,5-triphosphate receptor (IP_3_R) from the ER and also to incorporate extracellular calcium *via* the calcium-release-activated calcium channel (CRAC/ORAI) that is coupled with the stromal interaction molecule (STIM) in DT40 cells [31], [45], [46]. From this point of view, the function of ZIP9 may require intracellular calcium. The relationship between calcium and the ZIP9 function remains unclear. However, importantly, our data suggest that the increase in intracellular zinc level through ZIP9 is regulated by BCR activation without exogenous zinc, and coincides with the enhancement of Akt and Erk phosphorylation.

It has been reported that ZIP7 and ZIP13 that are located in the ER, the Golgi, or both increase the zinc influx into the cytosol in response to EGF/IGF and TGFß/BMP stimulations, respectively [12], [36], [47]–[49]. The function of ZIP7 has been also reported to be necessary to phosphorylate ZIP7 by CK2 in the human breast cancer cell line [13]. On the other hand, the regulation of zinc influx through ZIP13 by TGFß/BMP operates in the nuclear/cytosol shuttle of Smad2/3, which supply zinc into the mad-homology domain on Smad2/3 in primary dermal fibroblasts of mice [49]. More recently, a homodimer complex of ZIP13 is fundamental to the zinc influx [50]. Our findings reveal that the zinc release function of ZIP9 seems to be similar to that of ZIP7 and ZIP13. However, ZIP7 and ZIP13 are members of the LIV-1 subfamily and these two transporters are similar. Moreover, ZIP9 may have retained important function in chicken cells because the ZIP7 protein is not expressed in chicken [25]. Thus, the functional mechanism in mammalian or human cells, which is post-translational modification, or the higher-order structure of ZIP9 needs to be elucidated in further investigation.

**Figure 5:**
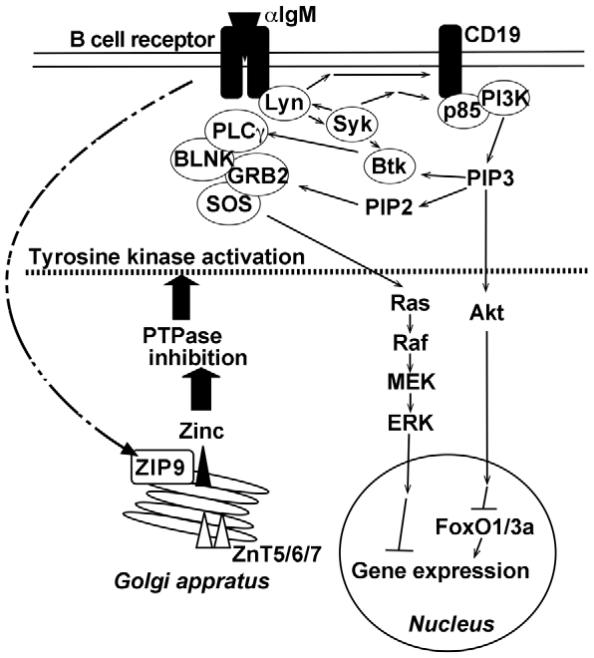
Proposed action sites of intracellular zinc release by ZIP9 in DT40 cells for activation of B cell receptor signaling. It is the proposed mechanism of Zn-induced PTPase inhibition by ZIP9, which leads to the activation of B cell receptor signaling in DT40 cells. Intracellular zinc is incorporated into the Golgi by ZnT5/6/7. Zinc is released as induced by ZIP9 into the cytosol from the Golgi, which in turn inhibits PTPase activity and induces the phosphorylation of Akt and ERK probably indirectly by regulating upstream components of the signal transduction.

Our findings indicate that the function of ZIP9 affects the level of cytosolic zinc, resulting in the activation of signaling kinase *via* PTPase inhibition in DT40 cells (Figure 5). As we have shown in this study, this is the first evidence for the function of the subfamily I of the ZIP family that could explain the relationship between the increase of intracellular zinc level by ZIP9 and BCR activation. These observations provide new mechanistic insights into signaling molecules and B-cell fate underlying the regulation of intracellular zinc level by ZIP9 in the response to antigen-stimulated BCR.
